# Downregulated pseudogene *CTNNAP1* promote tumor growth in human cancer by downregulating its cognate gene *CTNNA1* expression

**DOI:** 10.18632/oncotarget.10833

**Published:** 2016-07-25

**Authors:** Xiangjian Chen, Hua Zhu, Xiaoli Wu, Xuemeng Xie, Guanli Huang, Xiaoqun Xu, Shi Li, Chungen Xing

**Affiliations:** ^1^ Department of General Surgery, The Second Affiliated Hospital of Soochow University, Suzhou, Jiangsu, P.R. China; ^2^ Department of General Surgery, The First Affiliated Hospital of Wenzhou Medical University, Wenzhou, Zhejiang, P.R. China; ^3^ Department of Obstetrics and Gynecology, The First Affiliated Hospital of Wenzhou Medical University, Wenzhou, Zhejiang, P.R. China; ^4^ Department of Gastroenterology, The First Affiliated Hospital of Wenzhou Medical University, Wenzhou, Zhejiang, P.R. China; ^5^ Department of Surgical Oncology, The First Affiliated Hospital of Wenzhou Medical University, Wenzhou, Zhejiang, P.R. China; ^6^ Operating room, The First Affiliated Hospital of Wenzhou Medical University, Wenzhou, Zhejiang, P.R. China; ^7^ Department of Urology, The First Affiliated Hospital of Wenzhou Medical University, Wenzhou, Zhejiang, P.R. China

**Keywords:** CRC, ceRNA, qRT-PCR, lncRNA, CCK-8

## Abstract

Accumulating evidence indicates that deregulation of cancer-associated pseudogene is involved in the pathogenesis of cancer. In the study, we demonstrated that pseudogene *CTNNAP1*, for the *CTNNA1* gene, was dysregulated in colorectal cancer and the degree of dysregulation was remarkably associated with tumor node metastasis (TNM) stage (*P*<0.05). The mechanistic experiments revealed that pseudogene *CTNNAP1* played a pivotal role in the regulation of its cognate gene *CTNNA1* by competition for microRNA-141. Moreover, gain-of-function approaches showed that overexpression of *CTNNAP1* or *CTNNA1* significantly inhibited cell proliferation and tumor growth *in vitro* and *in vivo* by inducing G0/G1 cell cycle arrest. Our findings add a new regulatory circuit via competing endogenous RNA (ceRNA) cross-talk between pseudogene *CTNNAP1* and its cognate gene *CTNNA1*, and provide new insights into potential diagnostic biomarker for monitoring human colorectal cancer.

## INTRODUCTION

Colorectal cancer (CRC) is the third most commonly diagnosed malignancy and the leading cause of cancer-related deaths in the world [1]. In fact, throughout the last few decades, epidemiological studies has shown that multiple environmental factors, genetic or epigenetic abnormalities are involved in the initiation ad progression of CRC [2, 3]. Despite recent advances in diagnostic techniques and medical treatment, the overall survival of CRC patients remains still relatively low. Therefore, it is urgently needed to investigate the detailed pathophysiological mechanisms contributing to CRC which provide fundamental information for early diagnosis and treatment of CRC.

Lately, advances in the analysis of whole-genome sequencing data have showed that most genomic sequences is transcribed as non-coding RNA species, including long non-coding RNA (lncRNAs), pseudogenes and microRNAs etc. Numerous studies demonstrate that these non-coding transcripts are implicated in regulation of various cellular processes [4–7]. In recent studies, pseudogenes, which were recognized as a new class of non-coding RNAs, have been discovered sharing similar nucleotide sequence with their parental protein-coding genes [8]. However, these special genes lost their ability to produce functional protein products mostly arising as a consequence of premature stop codons or disabling mutations [9]. Similar to other non-coding RNAs, pseudogenes have been discovered to exert important roles in a variety of biological processes and human diseases, particularly in tumorigenesis [10–13]. To date, increasing evidence indicates that pseudogenes can act as competing endogenous RNA (ceRNA) to sustain the expression of their parental genes by competing for the binding of some of the same microRNA molecules [14]. As an example, the first ceRNA *PTENP1* sequesters microRNAs (microRNA-21, microRNA-19b and microRNA-20a) away from its mRNA target *PTEN*, thereby influencing parent gene expression [15]. In the recently reported study by Yang W and colleagues [16], *Foxo3* pseudogene (*Foxo3P*) could suppress tumor growth and angiogenesis by functioning as a sponge for microRNAs, and upregulate expression of the forkhead family of transcription factors, *Foxo3*.

In the present study, we investigated that pseudogene *CTNNAP1* was aberrantly expressed in CRC and was positively associated with *CTNNA1* expression. Furthermore, gain-of-function assays were further explored that pseudogene *CTNNAP1* could act as a ceRNA to increase *CTNNA1* gene expression through competition for microRNA-141, subsequently inhibiting cell proliferation and tumor growth. This study showed the first evidence for the cross-talk between *CTNNAP1* and *CTNNA1* via competing for microRNA-141, shedding a better understanding of molecular etiology of CRC.

## RESULTS

### *CTNNAP1* was downregulated in CRC

**Figure 1:**
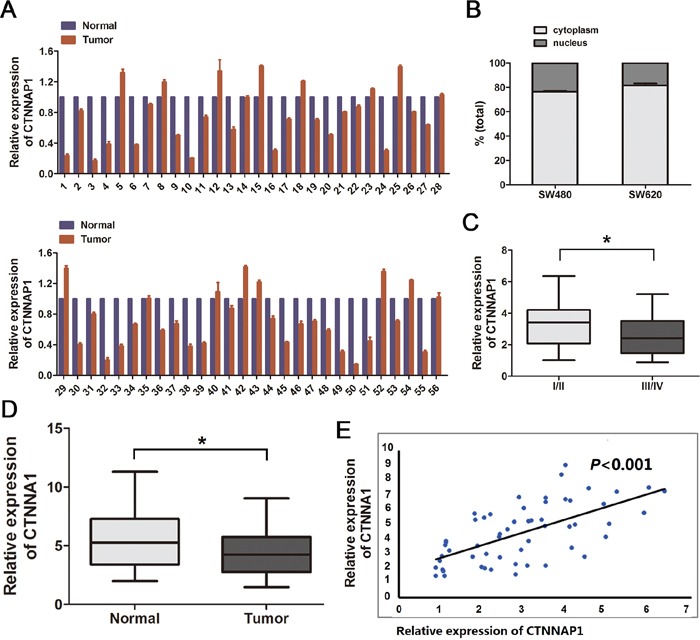
Expression levels of pseudogene CTNNAP1 and its cognate gene CTNNA1 in CRC **A.** Relative expression of pseudogene *CTNNAP1* in a cohort of 56 pairs of CRC tissues and paired nontumor tissues by real-time PCR. Data presented in tumor tissues is normalized to normal tissues. *P*<0.001, paired *t*-test. **B.** RNA of nuclear and cytoplasmic fractions in CRC cell line SW480 and SW620 was determined by qRT-PCR. *CTNNAP1* was mostly located in the cytoplasmic fractions. Mean±S.D. are shown (n=3). **C.** *CTNNAP1* expression was significantly downregulated in CRC patients with advanced TNM stage (III and IV) than those with low TNM stage (I and II). *P<0.05. **D.** *CTNNA1* is downregulated in CRC tissues compared with adjacent normal tissues (*P*<0.05, paired *t*-test). **E.** The expression of *CTNNAP1* is positively correlated with CTNNA1 in CRC tissues (*P*<0.001; R^2^=0.3987). The data were obtained by the logistic regression analysis.).

As an intriguing class of lncRNAs, recent evidence increasingly discovered that pseudogenes have crucial roles in normal physiology as well as quite recently in the context of cancer. To evaluate the expression of pseudogene *CTNNAP1*, we performed qRT-PCR assay in a cohort of 56 pairs of CRC tissues and paired nontumor tissues. The result showed that the expression of *CTNNAP1* was downregulated in 70% tumor samples (39/56) compared to adjacent normal samples (*P*<0.05; Figure 1A). Additionally, *CTNNAP1* subcellular localization was further analyzed in CRC cell lines. As showed in Figure 1B, *CTNNAP1* was predominantly detectable in the cytoplasm (more than 75 %) than in the nucleus of fractionated SW480 and SW620 cells.

We then sought to determine the correlation of *CTNNAP1* expression with clinicopathological features of CRC patients to assess its clinical significance. According to the median value (0.68) of relative *CTNNAP1* expression in CRC tissues, 56 CRC patients were classified into high group (n=28, *CTNNAP1* expression ratio>0.68) and low group (n=28, *CTNNAP1* expression ratio<0.68). We found that *CTNNAP1* expression levels in CRC tissues were remarkably associated with tumor node metastasis (TNM) staging (*P*<0.05; Table 1). More importantly, CRC patients with advanced TNM stage (III and IV) exhibited decreased *CTNNAP1* expression than those with low TNM stage (I and II) (*P*<0.05; Figure 1C).

**Table 1:** The correlation between *CNTTA1* and *CNTTAP1* expression and the clinical pathological factors of colorectal cancer patients

Parameters	Total (N)	Expression of *CNTTA1*				*P*_value_*	Expression of *CNTTAP1*				*P*_value_*
		High		Low			High		Low		
**Gender**											
male	28	15	26.79	13	23.21	0.592	12	21.43	16	28.57	0.285
female	28	13	23.21	15	26.79		16	28.57	12	21.43	
**Age**											
<50	22	12	21.43	10	17.86	0.584	13	23.21	9	16.07	0.278
≥50	34	16	28.57	18	32.14		15	26.79	19	33.93	
**Differentiation**											
High	16	11	19.64	5	8.93	0.706	10	17.86	6	10.71	0.707
Middle	28	9	16.07	19	33.93		11	19.64	17	30.36	
Low	12	8	14.29	4	7.14		7	12.5	5	8.93	
**Lymph node metastasis**											
No	30	18	32.14	12	21.43	0.109	17	30.36	13	23.21	0.284
Yes	26	10	17.86	16	28.57		11	19.64	15	26.79	
**Tumor size**											
<2cm	25	12	21.43	13	23.21	0.788	10	17.86	15	26.79	0.179
≥2cm	31	16	28.57	15	26.79		18	32.14	13	23.21	
**TNM stages**											
I+II	29	20	35.71	9	16.07	0.003*	19	33.93	10	17.86	0.016*
III+IV	27	8	14.29	19	33.93		9	16.07	18	32.14	

* Chi-square test

* *P*<0.05

We further evaluated the cognate gene *CTNNA1* of pseudogene *CTNNAP1* expression in CRC clinical samples. *CTNNA1* expression level is remarkably lower in CRC tissues in comparison with matched normal tissues (Figure 1D), and its expression is positively correlated with pseudogene *CTNNAP1* expression level (*P*<0.001, R^2^=0.399) (Figure 1E). Taken together, these analyses indicated that *CTNNAP1* may be a potential predictor for CRC development and progression.

### MicroRNA-141 inhibited pseudogene *CTNNAP1* and its cognate gene *CTNNA1* in CRC

**Figure 2:**
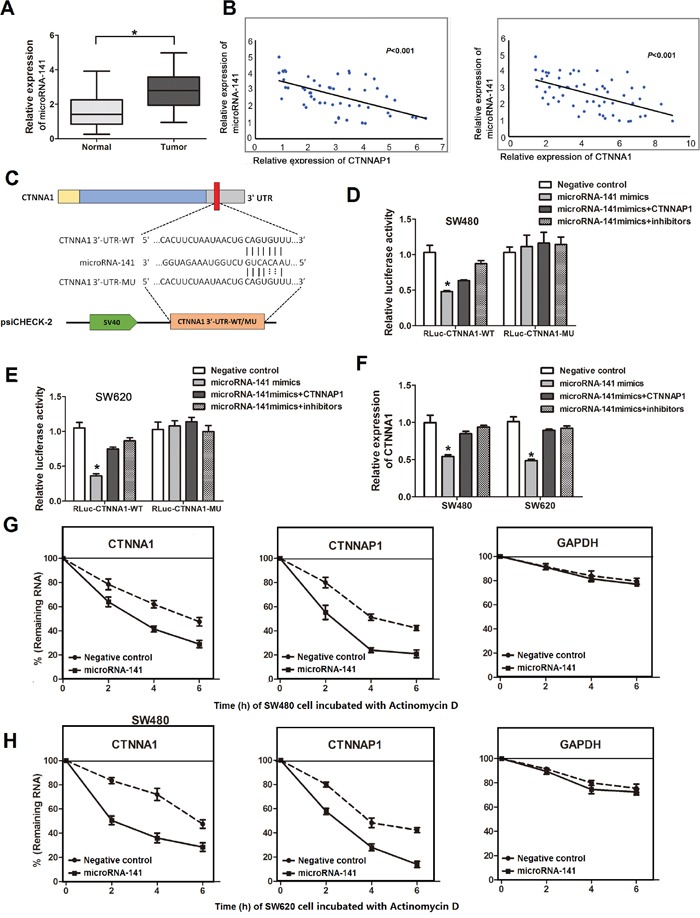
MicroRNA-141 inhibits the expression levels of pseudogene *CTNNAP1* and its cognate gene *CTNNA1* in CRC **A.** The expression of microRNA-141 in CRC tissues and paired nontumor tissues. The expression of microRNA-141 is significantly up-regulated in cancer tissues than normal controls. *P<0.05, paired *t*-test. **B.** Negative correlation between *CTNNAP1*, *CTNNA1* expression and microRNA-141 level in tissues of 56 CRC patients (*P*<0.001, R^2^=0.3166 between *CTNNAP1* and microRNA-141; *P*<0.001, R^2^=0.3038 between *CTNNA1* and microRNA-141). The data were obtained using the logistic regression analysis. **C.** Schematic outlining of human *CTNNA1* 3′-UTR, and the position of the predicted microRNA-141 binding sites on *CTNNA1* 3′-UTR sequence. The wild-type *CTNNA1* 3′-UTR containing the microRNA-141 recognition site (*CTNNA1* 3′-UTR-WT) or mutant *CTNNA1* 3′-UTR harboring mutated microRNA-141 binding site (*CTNNA1* 3′-UTR-MU) was cloned downstream of the luciferase gene. **D.** and **E.** Luciferase reporter containing the wild type *CTNNA1* 3′-UTR or mutant *CTNNA1* 3′-UTR were transfected plasmid harboring full length *CTNNAP1* (pcDNA3.1-*CTNNAP1*) or microRNA-141 inhibitors in combination with microRNA-141 mimics. The luciferase activity was determined by luciferase reporter assays. Data are mean±SD (n=3). *P<0.05, Two-side Student's *t*-test. **F.** After transfection microRNA-141 mimics with plasmid harboring full length *CTNNAP1* or microRNA-141 inhibitor, the effect of microRNA-141 on *CTNNA1* mRNA level or *CTNNAP1* in antagonizing microRNA-141-mediated suppression of *CTNNA1* mRNA level was examined by qRT-PCR. The data are presented as the mean±SD. *P<0.05, Two-side Student's *t*-test. Twenty-four hours after SW480 cells **G.** and SW620 cells **H.** were transfected with microRNA-141 mimics, the half-life of *CTNNAP1* and *CTNNA1* was measured using qRT-PCR. The data represent the Mean±SD from three independent experiments.

Pseudogenes are believed quite recently to play important roles in varies of diseases via competing for the binding of common microRNAs molecule with their parental genes, thereby liberating mRNA transcripts expression of microRNAs targets [17, 18]. In addition, since the positive expression trend between pseudogene *CTNNAP1* and its cognate gene *CTNNA1*, we further determined whether *CTNNAP1* can regulate the expression of *CTNNA1* through operating as a ceRNA. Based on the bioinformatics tools and the reference [11], 4 potential microRNAs binding sites scattered the *CTNNAP1* transcript as well as the sequence of *CTNNA1* 3′-UTR (microRNA-141, microRNA-18b, microRNA-33a and microRNA-9). Among these microRNAs, microRNA-141 was found to be up-regulated in the same CRC tissues in comparison with matched normal tissues (Figure 2A). Notably, microRNA-141 had been reported to promote cell growth, cell cycle progression and tumor invasion in CRC [19]. In addition, correlation analyses revealed that microRNA-141 significantly correlated with the expression of *CTNNAP1* and *CTNNA1* in the CRC tissues (*P*<0.001, R^2^=0.317 for *CTNNAP1*; *P*<0.001, R^2^=0.304 for *CTNNA1*) (Figure 2B).

Considering the potential binding sites for microRNA-141 in *CTNNAP1* and *CTNNA1* genes (Supplementary Table S1) as well as the coordinated expression levels of *CTNNAP1*, *CTNNA1* and microRNA-141, we performed dual luciferase reporter assays to investigate whether *CTNNAP1* and *CTNNA1* were regulated by microRNA-141. Reporter plasmids containing 3′-UTR of *CTNNA1* (RLuc-*CTNNA1*-WT or RLuc-*CTNNA1*-MU) (Figure 2C), which contains wild-type or mutant microRNA-141 binding sites transfected with microRNAs mimics or negative controls into CRC cells. The result showed that luciferase activity from the RLuc-*CTNNA1*-WT were significantly reduced by 47% and 35% in SW480 and SW620 cells compared with the negative controls (Figure 2D and 2E). Furthermore, reporter plasmids containing the wild type 3′-UTR of *CTNNA1* were subsequently transfected plasmid encoding *CTNNAP1* (pcDNA3.1-*CTNNAP1*) or microRNA-141 inhibitors along with microRNAs mimics. Expression of *CTNNAP1* and knockdown of microRNA-141 partially abrogated the inhibitory effect of microRNA-141 (Figure 2D and 2E). As we expected, luciferase activity of reporter plasmids containing the mutant *CTNNA1* 3′-UTR was not affected in cells which were transfected microRNA-141 mimics with inhibitors or plasmid encoding *CTNNAP1* in comparison with controls (Figure 2D and 2E), suggesting a direct interactions between microRNA-141 and its putative recognition sites. Subsequent qRT-PCR analysis further showed that overexpression of microRNA-141 in SW480 and SW620 cells decreased the expression of *CTNNA1* mRNA than the controls, whereas the inhibitory effect of microRNA-141 on *CTNNA1* expression was completely abolished by the introduction of *CTNNAP1* and knockdown of microRNA-141 (Figure 2F). Together, these data indicate that pseudogene *CTNNAP1* can function as microRNA-141 decoy, thereby increasing its cognate gene *CTNNA1* expression by sequestering microRNAs.

### The half-life of *CTNNAP1* and *CTNNA1* decreased by microRNA-141

We measured the *CTNNAP1* and *CTNNA1* half-life after inhibiting transcription by incubating cells with actinomycin D using qRT-PCR. As showed in Figure 2G and 2H, the transcript levels of the *CTNNAP1* and *CTNNA1* was declined in CRC cells after RNA synthesis was blocked with Actinomycin D in the presence of microRNA-141. Furthermore, the half-life of *CTNNAP1* and *CTNNA1* regulated by microRNA-141 was shorter in CRC cells (t_1/2_=2h for *CTNNAP1* and t_1/2_=4h for *CTNNA1* in SW480 cells; t_1/2_=3h for *CTNNAP1* and t_1/2_=2h for *CTNNA1* in SW620 cells) after actinomycin D treatment than in control cells (t_1/2_=5h for *CTNNAP1* and t_1/2_=6h for *CTNNA1* in SW480 cells; t_1/2_=4h for *CTNNAP1* and t_1/2_=5h for *CTNNA1* in SW620 cells). These results indicate that microRNA-141 could suppress *CTNNAP1* and its cognate gene *CTNNA1*.

### Effects of pseudogene *CTNNAP1* and its cognate gene *CTNNA1* on cell proliferation *in vitro*

**Figure 3:**
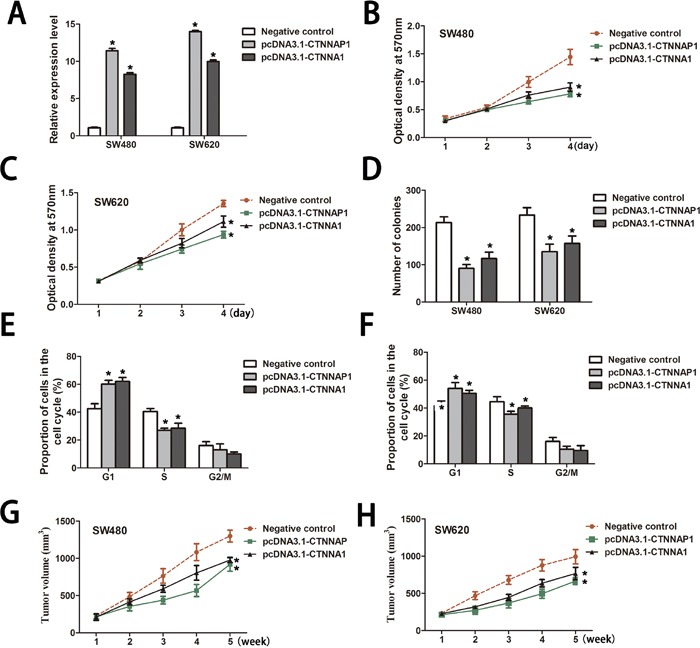
*CTNNAP1* and *CTNNA1* exert tumor suppressive effects on CRC cells *in vitro* and *in vivo* **A.** *CTNNAP1* and *CTNNA1* expression levels were confirmed by qRT-PCR in SW480 cells and SW620 cells. Mean±SD are shown (n=3). *P<0.05, Two-side Student's *t*-test. Upregulation of *CTNNAP1* and *CTNNA1* attenuated CRC cancer cell lines SW480 **B.** and SW620 **C.** proliferation at day 4 as determined by CCK8 assay (*P*=0.002 for *CTNNAP1* and *P*=0.004 for *CTNNA1* in SW480 cells; *P*<0.001 for *CTNNAP1* and *P*=0.008 for *CTNNA1* in SW620 cells). *P<0.05 compared with the control, Two-sided Student's *t*-test; n=6. **D.** After two weeks, colony formation of SW480 cells and SW620 cells was significantly suppressed by overexpressing *CTNNAP1* (*P*<0.001 in SW480 cells; *P*=0.004 in SW620 cells) or *CTNNA1* (*P*=0.002 in SW480 cells; *P*=0.01 in SW620 cells) when compared to the negative controls. The data are shown (mean±SD). *P<0.05 compared with the control, Two-sided Student's *t*-test; n=3. **E.** and **F.** Cell cycle phases of SW480 cells and SW620 cells with *CTNNAP1* or *CTNNA1* overexpression were determined by Flow cytometry (*P<0.05, Two-sided Student's *t*-test; n=3). **G.** and **H.** At week 5, the tumor volumes were dramatically smaller in nude mice injected with *CTNNAP1*-overexpressed or *CTNNA1*-overexpressed CRC cells compared to nude mice injected with negative control cells. Tumor volumes were determined every three days. The data are presented as the mean±SD.*P<0.05, Two-sided Student's *t*-test; n=6.

Pseudogene *CTNNAP1*, for the human alpha E-catenin *CTNNA1* gene, was originally characterized by fluorescence *in situ* hybridization [20]. Several studies have assessed the tumor suppressive role of *CTNNA1* in various tumors [21–24]. However, no studies have been conducted on the effects of *CTNNA1* gene on the progression of CRC. To elucidate the functions of *CTNNAP1* and *CTNNA1* in CRC, a series of functional assays were performed to investigate the roles of *CTNNAP1* and *CTNNA1* in cell proliferation and tumor growth in SW480 and SW620 cells. pcDNA3.1-*CTNNAP1* or pcDNA3.1-*CTNNA1* were transfected into SW480 and SW620 cells, respectively, and the transfection efficiency of *CTNNAP1* and *CTNNA1* overexpression were subsequently confirmed by qRT-PCR analysis. After 48h post-transfection, the RNA levels of *CTNNAP1* and *CTNNA1* revealed that *CTNNAP1* expression was increased by 11-fold and 13-fold in SW480 and SW620 cells than the empty vector pcDNA3.1, respectively (Figure 3A). Similar to *CTNNAP1*, relative level of *CTNNA1* was significantly up-regulated by 8-fold and 10-fold in SW480 and SW620 cells than the empty vector pcDNA3.1, respectively (Figure 3A).

Subsequently, we measured the effects of *CTNNA1* or *CTNNAP1* ectopic expression on cell proliferation. The CCK-8 assay showed that the increased expression of *CTNNA1* in CRC cells inhibited proliferation compared with the controls at day 4 (Figure 3B and 3C). Moreover, upregulation of *CTNNAP1* similarly decreased cell growth in SW480 and SW620 cells (Figure 3B and 3C). Accordingly, the overexpression of *CTNNA1* significantly suppressed the colony numbers of the SW480 and SW620 cells compared with the controls (Figure 3D). A similar effect of *CTNNAP1* overexpression on colony formation ability was also observed in a parallel with *CTNNA1* in SW480 and SW620 cells (Figure 3D). Furthermore, cell-cycle progression of transfected SW480 and SW620 cells was measured by using flow cytometry. As shown in Figure 3E and 3F, overexpression of either *CTNNAP1* or *CTNNA1* caused a cell-cycle arrest, with a significant increase in the proportion of cells in the G0/G1 phase compared with controls in the SW480 cells. Similar results were also observed in SW620 cells.

### The *CTNNA1* as well as *CTNNAP1* inhibited tumorigenesis of CRC *in vivo*

We further investigated the effects of overexpression of either *CTNNA1* as well as *CTNNAP1* on tumor growth *in vivo*. The CRC cells transfected with pcDNA3.1-*CTNNA1* or pcDNA3.1-*CTNNAP1* were injected subcutaneously into female nude mice. Five weeks after injection, tumors derived from *CTNNA1*-overexpression CRC cells were significantly smaller than those derived from empty vector-transfected cells (976.5±33.2mm3 versus. 1299.0±79.2mm3 for SW480; 766.3±83.3mm3 versus. 993.3±96.3mm3 for SW620) (Figure 3G and 3H). In addition, up-regulation of *CTNNAP1* in CRC cells induced a similar and smaller tumor size compared with controls (920.5±95.5mm3 versus. 1299.0±79.2mm3 for SW480; 666.0±46.9mm3 versus. 993.3±96.3mm3 for SW620) (Figure 3G and 3H). These results showed that *CTNNAP1* as well as *CTNNA1* could obviously inhibit CRC tumorigenesis *in vivo*. Taken together, the ability of *CTNNA1* and *CTNNAP1* to suppress cell proliferation and tumor growth indicates that *CTNNAP1* and its cognate gene *CTNNA1* may potentially play tumor suppressive roles in CRC.

## DISCUSSION

**Figure 4:**
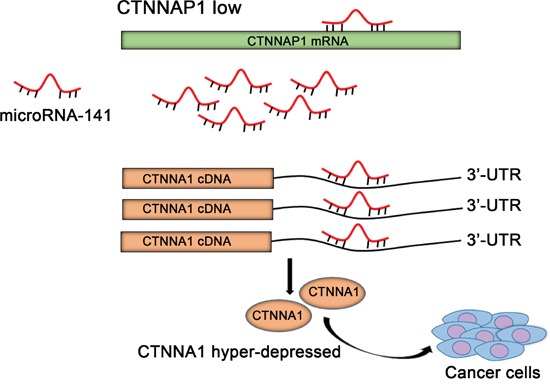
Schematic overview of pseudogene *CTNNAP1* regulatory network in CRC pathogenesis Downregulation of pseudogene *CTNNAP1* downregulated its cognate gene *CTNNA1* mRNA level via mediating microRNA-141 repression activity, thereby conferred a malignant phenotype to colorectal cancer cells.

Pseudogenes, as a large component of the human transcriptome, have long been neglected and considered as “junk” DNA [25]. Recently, most of the known pseudogenes have been extensively studied in normal physiology as well as in multiple cancer types [26, 27]. A growing body of evidence have revealed that dysregulation of pseudogenes may regulate the expression of *oncogenes or tumor*-suppressor genes by acting as modulators of microRNAs [10, 15, 28]. In the current study, we demonstrated for the first time that lower expression of pseudogene *CTNNAP1* resulted in *CTNNA1* mRNA level suppression by microRNA-141, and conferred a malignant phenotype to colorectal cancer cells lines (Figure 4).

Pseudogene *CTNNAP1* is located to human chromosome 5q22, which was first found from a human genomic phage library. Its cognate gene *CTNNA1* plays a central role in cell-cell contact by interacting with cadherin-catenin complex [29]. Clinical observations have intensively revealed the crucial role of *CTNNA1* in tumors [23, 30, 31]. However, as a pseudogene for a member of the E-cadherin/catenin complex, it remains unclear whether pseudogene *CTNNAP1* has important biological functions. In this study, we found that *CTNNAP1* was significantly downregulated in human CRC tissues and patients with lower *CTNNAP1* expression levels was significantly correlated with advanced pathological stage. These data imply that pseudogene *CTNNAP1* may emerge as a novel player in the development and progression of CRC. To further understand the biological functions of *CTNNAP1*, we conducted a series of functional experiments to determine the roles of *CTNNAP1* in CRC development. Inhibited cell proliferation and tumor growth were observed in *CTNNAP1*-overexpressed CRC cells. We next put the spotlight on the *CTNNAP1* expression influenced tumor-like characteristics, such as cell cycle progression. Our experiments showed that up-regulation of *CTNNAP1* in CRC cells led to a significant G1-G0 arrest and a related decrease in S phase. These findings indicate that the proliferation-inhibition effects of *CTNNAP1* in CRC probably result from the suppression of the G1-S phase transition.

Further investigating the molecular mechanism through which *CTNNAP1* led to the inhibition of CRC cell proliferation and tumor growth *in vitro* and *in vivo*. Pseudogene *CTNNAP1* exhibits 90% sequence identity to *CTNNA1*, which is believed to be important in mediating the linkage between the adhesion molecules E-cadherin and the actin cytoskeleton [32]. The abnormal assembly and expression of E-cadherin-catenin complex would break cell-cell adhesion, resulting in intravasation of primary cancer cells and enhancement of metastases formation [22, 33]. Accumulating evidences have assessed the expression levels of *CTNNA1* mRNA in a variety of cancers [34]. In this study, we found that *CTNNA1* expression was downregulated in CRC and positively correlated with that of *CTNNAP1*. Consistently, the functional studies *in vitro* and *in vivo* also verified the tumor suppressive roles of *CTNNA1* or *CTNNAP1* in CRC carcinogenesis. In addition, qRT-PCR analysis showed that microRNA-141 expression was inversely correlated with *CTNNA1* and *CTNNAP1* expression. In recent years, it has been discovered that microRNA-141 can influence *DLC1* and *SIP1* genes to participate in human diseases, including CRC [35–37]. And in the present study, we showed that *CTNNAP1* and *CTNNA1* are the major direct target genes of microRNA-141, though the results are not completely consistent with previous studies. Finally, the mechanisms accounting for the correlation expression of *CTNNAP1* and *CTNNAP1* showed that *CTNNAP1* behaved as a ceRNA to sustain the expression of its parental gene *CTNNA1* transcript from being inhibited by microRNA-141. Thus, *CTNNAP1* might be a promising candidate target for monitoring CRC.

In summary, the present study has suggested pseudogene *CTNNAP1* is a potential tumor suppressor participating in CRC pathogenesis by competing with the parent gene *CTNNA1* for microRNA-141. These findings shed a light on the potential of the regulatory network for investigating the underlying mechanisms of CRC pathogenesis and provided a valuable marker for the monitor of CRC.

## MATERIALS AND METHODS

### Tissue collection

A cohort of 56 CRC patients aged 18–78 years undergoing surgery at the First Affiliated Hospital of Wenzhou Medical University (Wenzhou, China) were enrolled, and written informed was obtained from each subject. The patient who received chemotherapy or radiotherapy prior to surgery were excluded. Clinical characteristics including age, sex, lymph node metastasis, tumor differentiation and TNM stage are shown in Table 1. This study was approved by Ethics Committee of Wenzhou Medical University.

### Cell lines

Human CRC cell lines (SW480 and SW620) were purchased from the American Type Culture Collection (USA). These cell lines were cultured routinely in RPMI Medium 1640 (Invitrogen) supplemented with 10% fetal bovine serum (Invitrogen, Shanghai, China) and were grown in incubator at 37°C with 5% CO_2_.

### Subcellular fractionation

For subcellular fractionation experiments, cytosolic and nuclear extracts from CRC cells (SW480 and SW620) were collected using a Nuclear/Cytosol Fractionation kit (Biovision) as previously described [38].

### Plasmid constructs and cell transfection

The cDNAs sequence of *CTNNAP1* and *CTNNA1* were synthesized and then subcloned into pcDNA3.1 (Invitrogen, Shanghai, China). The microRNA mimics, and microRNA inhibitors were from GenePharma (Shanghai, China). The stable CRC cells with ectopic expression of *CTNNAP1* or *CTNNA1* were achieved based on previously described method [39]. The empty vector was used as a control. Cells were harvested for quantitative real time RT-PCR (qRT-PCR) after 48h transfection using Lipofectamine 2000 (Invitrogen, Shanghai, China) according to the manufacturer's instructions.

### Actinomycin D assay

To measure half-life of CTNNAP1 and its cognate gene CTNNA1 regulated by microRNA-141. SW480 and SW620 cells were plated in 24-well culture plates. Twenty-four hours after cells were transfected with 40 pmol microRNA-141 mimics (Shanghai GenePharma Co., Ltd.), cells were incubated with Actinomycin D (Sigma) for 2, 4 or 6h. Actinomycin D was used at a final concentration of 2.5 mg/ml.

### QRT-PCR analyses

Total RNAs of tissues or cultured cells were extracted with TRIzol reagent (Invitrogen, Carlsbad, CA, USA) according to the manufacturer's protocol. The expression of mRNA was evaluated using SYBR Green Assays and microRNA expression was detected using Taqman microRNA Assays (Applied Biosystems) on ABI 7500 system (Applied Biosystems, CA, USA). The relative expression was normalized to the expression of glyc-eraldehyde-3-phosphate dehydrogenase (*GAPDH*) or *U6* using the 2^−ΔΔCt^. Each sample was analyzed in triplicate.

### Bioinformatics prediction and luciferase reporter assay

We used online software program TargetScan, starbase v2.0 and miRanda databases to predict potential microRNAs that have complementary base pairing with *CTNNAP1* and *CTNNA1* 3′-UTR. The sequence of *CTNNA1* 3′-UTR containing microRNAs putative target sites or *CTNNA1* 3′-UTR with point mutations in the microRNA response elements were amplified and then were cloned into psiCHECK-2 vector (promega). The vectors were cotransfected with microRNAs mimics or inhibitors into CRC cells using Lipofectamine 2000 (Invitrogen) for the reporter assay, according to the manufacturer's instructions.

### Cell proliferation assays

CRC cells transfected with pcDNA3.1-*CTNNAP1*, pcDNA3.1-*CTNNA1* or pcDNA3.1 empty vectors were collected and were plated in each well of a 96-well plate. Cell viability was measured every 24h by the Cell Counting Kit-8 (CCK-8) kit. For the colony formation assay, approximately 300 CRC cells transfected with pcDNA3.1-*CTNNAP1*, pcDNA3.1-*CTNNA1* or pcDNA3.1 empty vectors were plated into per well for six-well plates for 2 weeks incubation. The colonies were counted after fixing with methanol and staining with crystal violet (Sigma, USA) according to the manufacturer's instructions.

### Flow-cytometric analysis of cell cycle

These CRC cells transfected with pcDNA3.1-*CTNNAP1*, pcDNA3.1-*CTNNA1* or pcDNA3.1 empty vectors with overexpressed *CTNNAP1* or *CTNNA1* as described above were plated in six-well plates. After cultivation for 48h, the cells were harvested and subjected to analyze for cell cycle by a flow cytometer (FACSCalibur, BD Biosciences) according to the manufacturer's instructions. Each experiments was repeated three times independently.

### Xenograft studies

All female athymic BALB/c mice (5-week-old) were purchased from the Shanghai Experimental Animal Center of the Chinese Academy of Sciences and randomly were divided into control or experimental group. The animal study protocol was approved by the Animal Experimentation Ethics Committee of the Third Affiliated Hospital of Harbin Medical University. For a CRC mouse model system, 5×10^6^ CRC cell lines with overexpressed *CTNNAP1* or *CTNNA1* were injected subcutaneously in the posterior flank of BALB/c nude mice (6 mice per group). Tumor volumes were calculated every 3 days by measuring the length and width with calipers (Tumor volumes=0.5×length×width^2^).

### Statistical analysis

An unpaired two-tailed student's t-test and one-way analysis of variance (ANOVA) test were used to evaluate the significance of the differences. The expression relationship between *CTNNAP1*, *CTNNA1* and microRNAs in tissues was determined via linear regression model. Statistical analysis was performed using SPSS software (SPSS, Inc., Chicago, IL, USA). *P* values <0.05 was defined as statistically significant.

## SUPPLEMENTARY TABLE


