# Supplementary material for: Downregulated pseudogene CTNNAP1 promote tumor growth in human cancer by downregulating its cognate gene CTNNA1 expression
Source: Oncotarget. 2016 Jul 25;7(34):55518–28. doi: 10.18632/oncotarget.10833 (PMC5342433; doi:10.18632/oncotarget.10833)
Supplement: Supplementary file 1 [file oncotarget-07-55518-s001.pdf]

Downregulated pseudogene *CTNNAP1* promote tumor growth in human cancer by downregulating its cognate gene *CTNNA1* expression

SUPPLEMENTARY TABLE

Supplementary Table S1: The sequence of the predicted microRNA-141 binding sites in the region of *CTNNAP1* and *CTNNA1* 3’-UTR

Position	microRNA-141 binding site in *CTNNAP1*
616-639	5'-TTCAGCTGAAAGTTGTGGAAGATG-3'
2072-2093	5'-TCTTACTTTGAGATGGAAGATT-3'
2737-2766	5'-CTCAACCTTCCTGCTGTGTCATGGAAGATA-3'
**Position**	**microRNA-141 binding site in *CTNNA1* 3'-UTR**
703-724	5'-ACUUCUAAUAACUGCAGUGUUU-3'
